# Supplementary material for: Beam image-shift accelerated data acquisition for near-atomic resolution single-particle cryo-electron tomography
Source: Nat Commun. 2021 Mar 30;12:1957. doi: 10.1038/s41467-021-22251-8 (PMC8009872; doi:10.1038/s41467-021-22251-8)
Supplement: Supplementary file 3 — Description of Additional Supplementary Files [file 41467_2021_22251_MOESM3_ESM.pdf]

Description of Additional Supplementary Files

Supplementary Movie 1: Raw tilt-series of the tracking area obtained using BISECT. Sequence of 41 unaligned raw tilted projections collected over a ±60° tilt range acquired using BIS. We can achieve tracking with precision greater than 5 nm over the course of the tilt-series by using cross-correlation maximal distance thresholds of 50 nm at the low-magnification level, and 2 nm at the high-magnification level. Field of view is ~500 nm.

Supplementary Movie 2: Raw tilt-series of a ROI obtained using BISECT. Representative area situated 5.4 µm away in x along the tilt axis and 4.8 µm perpendicular to the tilt axis from the tracking area.
