# Supplementary material for: Beam image-shift accelerated data acquisition for near-atomic resolution single-particle cryo-electron tomography
Source: Nat Commun. 2021 Mar 30;12:1957. doi: 10.1038/s41467-021-22251-8 (PMC8009872; doi:10.1038/s41467-021-22251-8)
Supplement: Supplementary file 6 — Reporting Summary [file 41467_2021_22251_MOESM6_ESM.pdf]

nature research

Corresponding author(s): Mario J. Borgnia and Alberto Bartesaghi

Last updated by author(s): Feb 26, 2021

Reporting Summary

Nature Research wishes to improve the reproducibility of the work that we publish. This form provides structure for consistency and transparency in reporting. For further information on Nature Research policies, see our Editorial Policies and the Editorial Policy Checklist.

Statistics

For all statistical analyses, confirm that the following items are present in the figure legend, table legend, main text, or Methods section.

n/a	Confirmed	
☐	☒	The exact sample size (*n*) for each experimental group/condition, given as a discrete number and unit of measurement
☐	☒	A statement on whether measurements were taken from distinct samples or whether the same sample was measured repeatedly
☒	☐	The statistical test(s) used AND whether they are one- or two-sided<br>*Only common tests should be described solely by name; describe more complex techniques in the Methods section.*
☒	☐	A description of all covariates tested
☒	☐	A description of any assumptions or corrections, such as tests of normality and adjustment for multiple comparisons
☒	☐	A full description of the statistical parameters including central tendency (e.g. means) or other basic estimates (e.g. regression coefficient) AND variation (e.g. standard deviation) or associated estimates of uncertainty (e.g. confidence intervals)
☒	☐	For null hypothesis testing, the test statistic (e.g. *F*, *t*, *r*) with confidence intervals, effect sizes, degrees of freedom and *P* value noted<br>*Give P values as exact values whenever suitable.*
☒	☐	For Bayesian analysis, information on the choice of priors and Markov chain Monte Carlo settings
☒	☐	For hierarchical and complex designs, identification of the appropriate level for tests and full reporting of outcomes
☒	☐	Estimates of effect sizes (e.g. Cohen's *d*, Pearson's *r*), indicating how they were calculated

Our web collection on statistics for biologists contains articles on many of the points above.

Software and code

Policy information about availability of computer code

Data collection	SerialEM beta version 3.8.3 was used for data collection in combination with custom scripts that are available from https://gitlab.cs.duke.edu/bartesaghilab/bisect.
Data analysis	IMOD version 4.9, EMAN version 2.2, cisTEM version 1.0, CTFFIND4 version 4.1.12, and Phenix version 1.18.2 were used for data analysis.

For manuscripts utilizing custom algorithms or software that are central to the research but not yet described in published literature, software must be made available to editors and reviewers. We strongly encourage code deposition in a community repository (e.g. GitHub). See the Nature Research guidelines for submitting code & software for further information.

Data

Policy information about availability of data

All manuscripts must include a data availability statement. This statement should provide the following information, where applicable:

- Accession codes, unique identifiers, or web links for publicly available datasets
- A list of figures that have associated raw data
- A description of any restrictions on data availability

Cryo-EM density maps of dNTPase determined using BISECT/CSPT and regular SPA are deposited with the Electron Microscopy Data Bank (EMDB) with accession codes EMD-23355 and EMD-23356. The structure of the 80S ribosome obtained from EMPIAR-10064 has been deposited with accession code EMD-23357 and the structure of the E. coli 70S ribosome from EMPIAR-10304 with accession code EMD-23358. The atomic model of dNTPase is deposited in the Protein Data Bank (PDB) with accession code PDB ID 7LW5. All entries will be released immediately upon publication.

Field-specific reporting

Please select the one below that is the best fit for your research. If you are not sure, read the appropriate sections before making your selection.

- [x] Life sciences
- [ ] Behavioural & social sciences
- [ ] Ecological, evolutionary & environmental sciences

For a reference copy of the document with all sections, see nature.com/documents/nr-reporting-summary-flat.pdf

Life sciences study design

All studies must disclose on these points even when the disclosure is negative.

Sample size	The size of the tomographic dataset was chosen to ensure a minimum number of extracted particles of ~30,000, which is needed for high-resolution sub-tomogram averaging. The size of the single-particle dataset was chosen to match the number of particles in the tomographic dataset to ensure a meaningful comparison. All other sizes were determined by the amount of data deposited in the corresponding entry from the EMPIAR public repository.
Data exclusions	No sub-tomograms were excluded from the data analysis. High-tilt exposures were automatically down-weighted by our exposure compensation routines. Low quality tilt-series were excluded from the analysis based on their CTF estimation scores as described in the methods section.
Replication	The structures presented in this study were independently reproduced by three of the authors in this manuscript (one postdoc, one PhD student and one PI), producing maps of the same resolution each time.
Randomization	Randomization is not relevant for this study because no allocation into experimental groups was required (other than the separation into odd/even particle groups for the purpose of resolution estimation using the Fourier Shell Correlation criteria).
Blinding	Blinding was not relevant because no group allocations were required.

Reporting for specific materials, systems and methods

We require information from authors about some types of materials, experimental systems and methods used in many studies. Here, indicate whether each material, system or method listed is relevant to your study. If you are not sure if a list item applies to your research, read the appropriate section before selecting a response.

Materials & experimental systems

n/a	Involved in the study	
☒	☐	Antibodies
☒	☐	Eukaryotic cell lines
☒	☐	Palaeontology and archaeology
☒	☐	Animals and other organisms
☒	☐	Human research participants
☒	☐	Clinical data
☒	☐	Dual use research of concern

Methods

n/a	Involved in the study	
☒	☐	ChIP-seq
☒	☐	Flow cytometry
☒	☐	MRI-based neuroimaging
